# Recurrent intracranial hemorrhage without brain metastasis after VEGFR TKI rechallenge in metastatic renal cell carcinoma: a case report and literature review

**DOI:** 10.3389/fonc.2026.1858153

**Published:** 2026-06-08

**Authors:** Chang-Hoon Lee, Na-Ri Lee

**Affiliations:** 1 Division of Hematology and Oncology, Department of Internal Medicine, Jeonbuk National University Hospital, Jeonbuk National University Medical School, Jeonju, Republic of Korea; 2 Research Institute of Clinical Medicine, Jeonbuk National University, Biomedical Research Institute of Jeonbuk National University Hospital, Jeonju, Republic of Korea

**Keywords:** cerebellar hemorrhage, pazopanib, renal cell carcinoma, sunitinib, VEGFR tyrosine kinase inhibitor

## Abstract

Vascular endothelial growth factor receptor tyrosine kinase inhibitors (VEGFR TKIs) are widely used in the treatment of metastatic renal cell carcinoma (RCC). Although hemorrhagic events are recognized adverse effects of these agents, intracranial hemorrhage is rare, and the safety of re-administration of VEGFR TKIs after central nervous system (CNS) hemorrhage remains unclear. We report a 52-year-old man with metastatic clear cell RCC who initially received first-line immunotherapy with ipilimumab and nivolumab, resulting in disease progression, followed by second-line sunitinib, which achieved a partial response. However, he subsequently developed sudden neurological deterioration, and brain imaging revealed multifocal cerebellar hemorrhages without evidence of brain metastasis; the absence of metastasis was confirmed by surgical and histopathological examination. After surgical management, complete radiologic resolution of the hemorrhage was confirmed 4 weeks postoperatively. Pazopanib was subsequently initiated based on the prior favorable response to VEGFR TKI therapy; however, recurrent cerebellar hemorrhage occurred within 4 weeks of rechallenge, leading to rapid clinical deterioration and death despite supportive care. This case highlights that VEGFR TKI–associated intracranial hemorrhage can occur even in the absence of brain metastases and may recur with fatal consequences upon rechallenge. These findings suggest that early VEGFR TKI rechallenge (<1–2 months) after CNS hemorrhage may be unsafe, and that careful risk–benefit assessment with an adequate waiting period is essential when considering re-administration.

## Introduction

The management of metastatic renal cell carcinoma (RCC) underwent a major paradigm shift in the mid-2000s with the introduction of vascular endothelial growth factor receptor (VEGFR) tyrosine kinase inhibitors (TKIs). Since the approval of VEGFR TKIs in 2005, agents such as sunitinib and pazopanib have become standard systemic therapies for metastatic RCC (1–5). More recently, next-generation TKIs, including axitinib, cabozantinib, and lenvatinib, have also demonstrated important roles in the treatment of advanced RCC (6–9).

Hemorrhage and thrombosis are recognized vascular toxicities associated with VEGFR TKIs. VEGF plays a critical role in maintaining vascular integrity by promoting endothelial cell proliferation and survival. It also contributes to the coagulation cascade by inducing tissue factor expression in endothelial cells, thereby activating platelets and promoting the conversion of fibrinogen into fibrin (10, 11). Disruption of endothelial cell homeostasis caused by VEGFR inhibition may therefore result in hemorrhagic or thrombotic complications.

In clinical trials, most hemorrhagic events such as epistaxis were mild and manageable, and the reported incidence of intracranial hemorrhage was less than 1% (2–9). However, subsequent studies have suggested an increased risk of hemorrhagic events in patients with various cancers treated with VEGFR TKIs (12, 13). In addition, a significantly increased risk of intracerebral hemorrhage has been reported in patients with metastatic RCC and brain metastases receiving VEGFR TKI therapy (10).

Because treatment options for refractory or relapsed metastatic RCC remain limited, re-administration of VEGFR TKIs may be considered even after hemorrhagic complications. However, the safety and optimal timing of VEGFR TKI rechallenge following intracranial hemorrhage remain unclear. We report a fatal case of recurrent intracranial hemorrhage following VEGFR TKI rechallenge in a patient with metastatic RCC without brain metastasis, highlighting the potential risks associated with early re-administration.

## Case description

A 52-year-old man presented to the emergency department with altered mental status. He had experienced persistent vomiting for 2 days and, on the day of admission, responded only to painful stimuli.

One year earlier, he had been diagnosed with clear cell RCC of the left kidney with spinal metastases at T7 and L2, resulting in spinal cord compression. The patient underwent surgical resection of the spinal tumor followed by posterior spinal stabilization. Subsequently, palliative immunotherapy with ipilimumab (1 mg/kg) and nivolumab (3 mg/kg) was initiated and administered intravenously every 3 weeks. After three cycles of immunotherapy, imaging studies demonstrated disease progression with enlargement of renal and bone lesions and the appearance of new pulmonary metastases. The patient had complete paraplegia of the lower extremities prior to spinal surgery, which did not improve after surgery or with subsequent treatments.

Second-line palliative therapy with sunitinib (50 mg/day) was initiated using a 2-weeks-on/1-week-off schedule. After six cycles, a partial response was achieved: the renal and bone lesions decreased in size, and pulmonary metastases completely resolved. The patient had no significant past medical history and was not taking any medications before treatment. During sunitinib therapy, hypertension developed but was well controlled with controlled-release nifedipine (80 mg/day) and irbesartan (150 mg/day). He also experienced hand-foot syndrome and fatigue, which led to dose reduction to 37.5 mg/day after the sixth cycle.

After the eighth cycle of sunitinib, the patient was admitted with a sudden decline in consciousness. There was no history of head trauma or anticoagulant use. Upon examination, his blood pressure was 150/90 mmHg, pulse rate was 90 beats/min, respiratory rate was 20 breaths/min, and body temperature was 36.7 °C.

Laboratory evaluation revealed mild thrombocytopenia (platelet count 104 × 10³/μL), a white blood cell count of 4.4 × 10^9^/L, and a hemoglobin level of 13.9 g/dL. The coagulation profile, including prothrombin time and activated partial thromboplastin time, was within normal limits.

Brain magnetic resonance imaging (MRI) demonstrated multifocal hemorrhages in both cerebellar hemispheres, raising suspicion for hemorrhagic metastases (**Figures 1A–D**). Susceptibility-weighted imaging showed no disseminated strictly lobar microbleeds or cortical superficial siderosis to suggest cerebral amyloid angiopathy (**Figure 1C**). MR angiography showed no aneurysm, arteriovenous malformation, or vasculopathy. Because the hemorrhage occurred spontaneously, alternative etiologies were carefully considered. There was no history of head trauma, antiplatelet therapy, anticoagulant use, or known bleeding disorder. Emergency craniotomy was performed, revealing subacute hemorrhage without evidence of tumor. Histopathological examination demonstrated cerebellar tissue with red neurons and Rosenthal fibers, along with extensive hemorrhage but no malignant cells (**Figure 2**). The patient regained consciousness after surgery, and follow-up brain MRI 4 weeks later confirmed complete resolution of the cerebellar hemorrhage. (**Figures 1E–H**).

**Figure 1:**
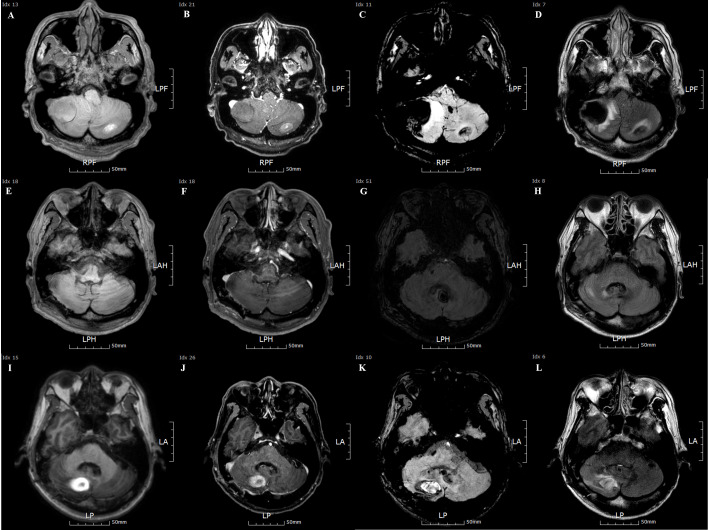
Brain MRI findings before and after hematoma evacuation. **(A)** Axial pre-contrast T1-weighted image, **(B)** axial post-contrast T1-weighted image, **(C)** axial susceptibility-weighted image, and **(D)** axial T2-weighted fluid-attenuated inversion recovery (T2-FLAIR) image demonstrating acute cerebellar hemorrhages measuring 4.5 × 3.2 cm in the right cerebellar hemisphere and 2 × 1 cm in the left cerebellar hemisphere. **(E)** Axial pre-contrast T1-weighted image, **(F)** axial post-contrast T1-weighted image, **(G)** axial susceptibility-weighted image, and **(H)** axial T2-FLAIR image obtained 4 weeks after hematoma evacuation, showing complete resolution of the cerebellar hemorrhage. Brain MRI obtained 4 weeks after initiation of pazopanib therapy. **(I)** Axial pre-contrast T1-weighted image, **(J)** axial post-contrast T1-weighted image, **(K)** axial susceptibility-weighted image, and **(L)** axial T2-FLAIR image demonstrating a 3.5 × 3 cm subacute hemorrhage in the right cerebellar hemisphere with surrounding edema.

**Figure 2:**
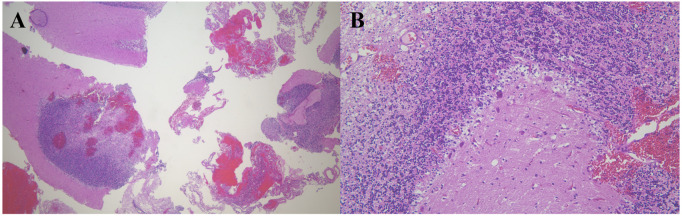
Histopathological findings of the cerebellar lesion. **(A)** Low-power view (H&E, ×40) showing cerebellar parenchyma with extensive hemorrhage. **(B)** High-power view (H&E, ×200) demonstrating hemorrhage and inflammatory cell infiltration without evidence of metastatic tumor cells.

Because the patient had previously achieved a substantial radiologic response to sunitinib, whereas immune checkpoint inhibitor therapy had resulted in disease progression, systemic treatment options were limited to another VEGFR TKI or mTOR inhibitors. Although mTOR inhibitors were considered because they are not typically associated with VEGFR-related hemorrhagic toxicity, concerns regarding their relatively modest efficacy in metastatic RCC and the possibility of insufficient tumor control influenced the treatment decision-making process. Given the patient’s prior favorable response to VEGFR inhibition and the need to consider a potentially more active therapeutic option, another VEGFR TKI was considered. In addition, because the patient had shown rapid postoperative recovery, there was an expectation that potential complications, if they occurred, might be manageable. After detailed discussion of the potential benefits and risks, including the possibility of recurrent hemorrhage, pazopanib was selected based on its relatively favorable tolerability profile compared with some alternative VEGFR TKIs.

Pazopanib (800 mg/day) was initiated 4 weeks after surgery. Hypertension recurred after initiation of pazopanib but was controlled with reintroduction of nifedipine and irbesartan. After 3 weeks of pazopanib therapy, the patient developed fatigue and generalized weakness, prompting dose reduction to 400 mg/day. However, 4 weeks after initiating pazopanib, he again developed altered mental status and nystagmus. Brain MRI revealed a new 3.5-cm subacute hemorrhage in the right cerebellar hemisphere (**Figures 1I–L**). At that time, his platelet count was normal (236 × 10³/μL), and his blood pressure was slightly elevated, at 160/89 mmHg.

Because the family declined further surgery and the neurosurgeon did not recommend reoperation, the patient received supportive care including valproic acid for seizure control. Unfortunately, the patient died of respiratory failure 8 days after the second cerebellar hemorrhage. A comprehensive clinical timeline of this case is provided in **Figure 3**.

**Figure 3:**
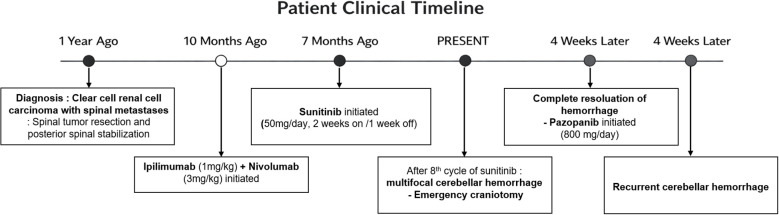
Timeline of clinical events and presentation.

## Discussion

Renal cell carcinoma is a heterogeneous group of malignancies arising from renal tubular epithelial cells. Although immune checkpoint inhibitors, such as nivolumab, ipilimumab, pembrolizumab, and avelumab, have expanded treatment options for RCC, VEGFR TKIs remain an important component of systemic therapy in metastatic disease.

Common adverse effects of VEGFR TKIs include hypertension, impaired wound healing, fatigue, diarrhea, and hypothyroidism, all of which are generally manageable. However, VEGFR TKIs are also associated with an increased risk of hemorrhagic complications such as epistaxis, hemoptysis, gastrointestinal bleeding, and central nervous system (CNS) hemorrhage.

Although clinical trials have reported a relatively low incidence of hemorrhagic complications, meta-analyses have demonstrated a significantly increased hemorrhagic risk in patients treated with VEGFR TKIs. VEGF promotes endothelial cell proliferation and survival, maintains vascular integrity, and increases nitric oxide production while inhibiting platelet activity and leukocyte adhesion. Inhibition of VEGF signaling may therefore impair vascular repair mechanisms and expose pro-coagulant phospholipids, leading to thrombosis or hemorrhage (10, 11, 14, 15).

A meta-analysis reported that the overall incidence of all-grade and high-grade hemorrhage was 9.1% and 1.3%, respectively, with hemoptysis (41.8%) and CNS hemorrhage (13.4%) being the most common high-grade events (12).

A review of previously reported cases of VEGFR TKI-associated CNS hemorrhage shows that most hemorrhages occurred at sites of pre-existing brain metastases (**Table 1**) (10, 17, 18, 20, 21). Importantly, two previously reported cases described VEGFR TKI-associated intracranial hemorrhage in the absence of brain metastases. Miller-Patterson et al. reported pazopanib-associated intracerebral hemorrhage accompanied by posterior reversible encephalopathy syndrome in a patient with RCC and uncontrolled hypertension (16). Similarly, Simao et al. described convexal subarachnoid hemorrhage during pembrolizumab–lenvatinib therapy in a patient without CNS metastases (19). These reports, together with our case, suggest that VEGFR TKI-associated CNS hemorrhage may occur even in non-metastatic settings, particularly in the presence of vascular vulnerability or hypertension.

**Table 1:** Published cases of central nervous system hemorrhage during VEGFR tyrosine kinase inhibitor therapy.

Authors	Drug	Age/Sex	Tumor type	Previous cardiovascular disease	History of hypertension	History of anticoagulant therapy	Brain metastasis/therapy	Hemorrhage site and type	Combination therapy	Duration of therapy	treatment	Survival duration of post bleeding	Other AEs
Pouessel et al. (10)	sunitinib	71/F	Renal cell carcinoma (clear cell)	None	None	None	None	ICH	None	14days	Conservative management	3days (death)	Hypertension
Pouessel et al. (10)	sunitinib	59/F	Renal cell carcinoma (clear cell)	None	None	None	Yes/None	ICH around the frontal BM	None	4days	Metastasectomy	3days (death)	None
Pouessel et al. (10)	sorafenib	75/M	Hepatocellular carcinoma	None	None	None	Yes/None	Intratumoral hemorrhage of Lt. frontal cortex	None	4days	Craniotomy	ND	ND
Pouessel et al. (10)	sorafenib	71/M	Renal cell carcinoma (papillary)	Cerebral stroke	None	Antiplatelet therapy	Yes/whole brain radiotherapy	ICH	None	2days	Conservative management	1day (death)	Hypertension
Pouessel et al. (10)	sorafenib	62/M	Renal cell carcinoma (clear cell)	None	Yes	None	Yes/whole brain Radiotherapy	ICH with subarachnoid bleeding	None	6days	Conservative management	3days (death)	Hypertension
Miller-Patterson et al. (16)	pazopanib	69/F	Renal cell carcinoma (clear cell)	None	Yes	ND	None	ICH of Rt.occipital lobe	None	3weeks	Craniectomy	ND	Hypertensive crisis. Posterior reversible encephalopathy syndrome
Chen et al. (17)	cabozantinib	57/F	Unknown primary cancer	None	ND	Ribaroxaban d/t DVT	Yes/None	Multifocal intratumoral bleeding	None 7days after Rivaroxaban d/t DVT	26months	Conservative management	Few days (death)	DVT
Sato et al. (18)	cabozantinib	72/F	Renal cell carcinoma	None	Yes	None	Yes/Stereotactic radiotherapy (35 Gy)	Intratumoral hemorrhage of Lt. parietal lobe	nivolumab	1week	Conservative management	12months (alive)	Hypertension (grade 1)
Simao et al. (19)	lenvatinib	60/F	Endometrial cancer	ND	ND	ND	None	bilateral convexal subarachnoid haemorrhage	pembrolizumab	ND	Conservative management	ND	ND
Iwasa et al. (20)	lenvatinib	56/F	Renal cell carcinoma (clear cell)	ND	ND	ND	Yes/Stereotactic radiotherapy (35 Gy)	Intratumoral hemorrhage of Rt. Insula cortex	pembrolizumab	30 days	Conservative management	1day (death)	Thrombocytopenia (grade 1)
Patel et al. (21)	Regorafenib	59/M	Colorectal adenocarcinoma	Congestive heart failure	Yes	ND	Yes/Surgery (left frontoparietal craniotomy and tumor resection)	ICH at and around the resection site, occurring 32 hours postoperatively	None	ND	Conservative management	ND	ND

ND, not described; DVT, deep vein thrombosis; ICH, intracerebral hemorrhage.

Hemorrhage most often developed within 2 days to 1 month after initiation of VEGFR TKI therapy.

The concomitant use of anticoagulants such as rivaroxaban for deep vein thrombosis may further increase the risk of CNS hemorrhage (17). In addition, inadequate discontinuation of regorafenib prior to surgery has been associated with postoperative CNS hemorrhage (21).

Hemorrhagic complications have also been reported in patients receiving VEGFR TKIs in combination with immune checkpoint inhibitors (18–20). Such combination therapy may theoretically increase hemorrhagic risk through endothelial dysfunction, impaired vascular stability, and alterations in intratumoral microvascular architecture.

One reported case involved a 72-year-old woman with RCC brain metastases treated with stereotactic irradiation (35 Gy in five fractions). CNS hemorrhage occurred 1 week after initiation of combined cabozantinib and nivolumab therapy at the metastatic site. The patient recovered with conservative corticosteroid treatment. Nivolumab monotherapy was resumed 1 month later and continued for 4 months. Cabozantinib was reintroduced 5 months after the initial hemorrhage due to progression of pulmonary metastases, with no recurrence of CNS hemorrhage during 6 months of follow-up (18). However, this case involved hemorrhage occurring at an irradiated metastatic brain lesion during combination therapy, representing a substantially different clinical context from non-metastatic cerebellar hemorrhage. Therefore, extrapolation of a specific VEGFR TKI rechallenge interval from a single case should be interpreted cautiously.

In our patient, CNS hemorrhage occurred 24 weeks after initiation of sunitinib, which was later than in most previously reported cases. The patient developed hypertension during sunitinib therapy, which was partially controlled (150/90 mmHg at the time of hemorrhage), along with mild thrombocytopenia (104 × 10³/μL). Brain MRI initially suggested hemorrhagic metastasis; however, craniotomy and pathological examination revealed no metastatic involvement, confirming non-metastatic cerebellar hemorrhage.

Because the patient had previously shown a favorable response to VEGFR TKI therapy and wished to continue systemic treatment, pazopanib, a VEGFR TKI reported to have a relatively favorable toxicity profile, was initiated 4 weeks after surgery following complete radiologic resolution of the initial hemorrhage. Unfortunately, CNS hemorrhage recurred 4 weeks after pazopanib initiation and ultimately resulted in the patient’s death.

Comparison with previously reported cases suggests that re-administration of VEGFR TKIs in this patient may have occurred too early. Given the limited evidence, no definitive recommendation regarding the optimal timing of VEGFR TKI rechallenge after CNS hemorrhage can currently be made. However, early rechallenge after recent CNS hemorrhage may warrant particular caution. Careful monitoring of blood pressure and platelet counts is essential, and caution should be exercised when antiplatelet or anticoagulant therapy is used concurrently.

Because VEGFR TKIs are known to increase bleeding risk, recurrent hemorrhage after early re-initiation in a recent post-ICH setting may not be entirely unexpected. However, this case is clinically relevant because fatal recurrent cerebellar hemorrhage occurred after rechallenge despite radiologic resolution of the initial hemorrhage and absence of brain metastasis.

## Conclusion

Recurrent intracranial hemorrhage is a rare but potentially fatal complication that may occur during VEGFR TKI therapy in metastatic RCC, even in the absence of brain metastasis. Because current evidence remains limited and most reported VEGFR TKI-associated CNS hemorrhages have occurred in patients with brain metastases or other bleeding risk factors, causality should be interpreted cautiously. Nevertheless, this case suggests that early VEGFR TKI rechallenge after recent CNS hemorrhage may carry substantial risk. When rechallenge is considered necessary, individualized risk–benefit assessment, careful neuroimaging evaluation, and close monitoring of blood pressure, platelet count, and concomitant medications are essential.

## Data Availability

The original contributions presented in the study are included in the article/supplementary material. Further inquiries can be directed to the corresponding author.
